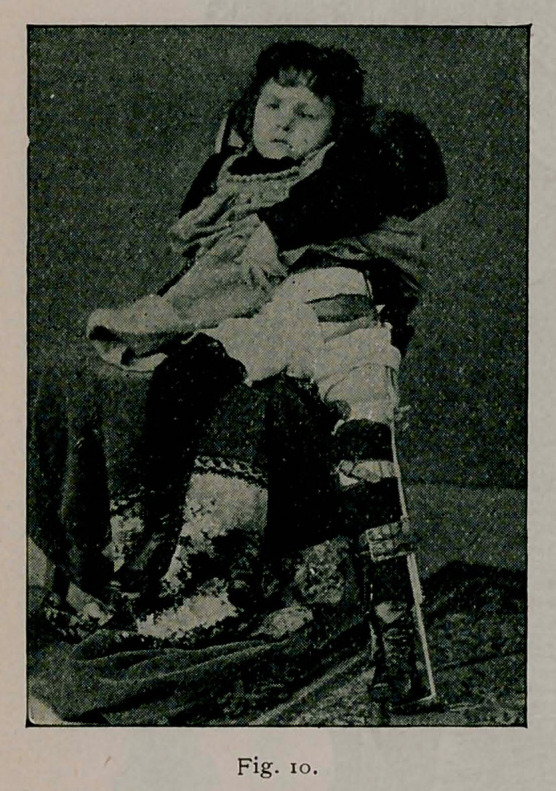# Tubercular Disease of the Spine and Hip and Early Symptoms of Hip-Joint Disease and Treatment1Read at the 26th annual meeting of the Alumni Association of the University of Buffalo Medical College, April 26, 1901.

**Published:** 1901-09

**Authors:** A. M. Phelps

**Affiliations:** New York City


### BUFFALO MEDICAL JOURNAL.

VOL. XLI.—LVII. SEPTEMBER, 1901. NO. 2.

### ORIGINAL COMMUNICATIONS.

### Tubercular Disease of the Spine and Hip and Early Symptoms of Hip-Joint Disease and Treatment.[1]

By A. M. PHELPS, A. M., M. D., New York City.

#### I.—POTT'S DISEASE.

THE name "Pott's disease of the spine and hip disease," in view of the light thrown upon the subject by our knowledge of modern pathology and surgical bacteriology, is a most unsatisfactory term. When the disease was first described by Pott, little was known of the etiology and pathology of the affection. He described the deformity of the spine which rapidly increased, producing a marked kyphosis, attended or unattended with abscess. This was supposed to be due to a constitutional taint. He recognised post mortem the breaking down of the bodies of the vertebræ, extensive destruction of the soft parts and of the bone, and this he knew was what produced the kyphosis. All cases presenting this characteristic deformity, attended or unattended with abscess, from that day to the present, have been termed "Pott's disease of the spine," and so Sayre described hip disease. Only a few years ago Mr. Bright, of England, published his remarkable paper on Bright's disease of the kidney. He recognised during life albumin and casts in the urine, and upon post mortem examination he found conditions of the kidney which produced it; therefore, until quite recently, when albumin and casts were found in the urine, the case has been termed "Bright's disease of the kidney." But careful investigation by pathologists has demonstrated that a great many

1. Read at the 26th annual meeting of the Alumni Association of the University of Buffalo Medical College, April 26, 1901.

conditions of the kidney produce this condition of the urine found in so-called Bright's disease, and, in fact, this condition of the urine may exist and the kidney present no structural change whatever. The investigations of pathologists and bacteriologists have gradually classified the different affections of the kidney, until today we seldom speak of Bright's disease of the kidney, excepting we know that actual degeneration has taken place, or where we use the term "acute Bright's," a pathological condition which we all recognise exists. So, in Pott's disease of the spine it should be our duty, as far as possible, to classify the affections which produce similar symptoms, and which are now all classified under the one name, "Pott's disease of the spine." The same is true of the hip.

We recognise at the present time tubercular disease of the bodies of the vertebræ—a disease beginning insidiously, progressing slowly, covering periods of months or even years, and resulting or not resulting in abscess. We also recognise another condition—acute osteomyelitis—in which the onset of the disease is sudden, its progress active and virulent, the destruction of the bone extensive, and the formation of abscess following very soon after the attack. A condition very closely resembling Pott's disease of the spine follows in the course of the eruptive fevers, particularly of typhoid. This is no doubt of an inoculation into the spinal vertebræ of matter which has been absorbed from the Pyerian patches of the intestine. This condition of the spine has its own peculiar symptoms, and is quite easily recognised. Then, another disease of the spine which closely resembles Pott's, a rare affection, is due to the inoculation of the germs of actinomycosis. A beautiful specimen of this is in the anatomical museum of the University of Vienna. So you see that, at least four diseases are now recognised, and can be demonstrated to produce symptoms similar to those described by Pott, and which are now being jumbled together and classified under the one name—Pott's disease of the spine.

Now, in regard to the etiology of these diseases of the spine and joint: undoubtedly the disease must be preceded by a localised inflammation. Into this area of inflammation are inoculated the germs which produce the destructive changes in the bodies of the vertebræ. That Pott's or hip disease is a manifestation of a "constitutional taint," I think is incorrect; that it is a localised focus of disease, I believe. To illustrate: germ life, to grow and produce its destructive changes, must have a soil fit for its reception and nutrition, and it is only within the area of the active process of repair in which large masses of embryonic cell tissue are present that we find such a soil. Unless there is a lesion of an inflammatory nature in any portion of the body, germ life cannot find a foothold for its growth. For example, an incised wound is made; we watch it closely and find that the first process that takes place is an effusion of blood and coagulation; then a rapid wandering of cells into this blood clot occurs, and rapid cell proliferation. These cells rapidly form themselves into line between the cut ends of the tissues for the purpose of repair. Very soon organisation takes place, new tissue is built up; loops of capillaries are thrown into these tissues. After this, contraction begins to take place, the capillaries are destroyed, the epithelium grows over the surface of the wound, and we say the wound is healed. This, I believe, to be the normal process of repair. This is inflammation, and this is as normal as the growth of the stag's horn. This reparative inflammation is necessary in all cases where injury has been inflicted. If at any time during this process of repair or normal inflammation, germ life is inoculated into this new inflammatory tissue, another condition is at once established. If these germs are streptococci or some one of the pyogenic germs, they at once seize upon this new inflammatory material, and they with their ptomaines destroy it. In this case inoculation has taken place and we say that the wound has become infected, and disease is the result. This disease is suppuration.

If the germs of tuberculosis should be inoculated into this new inflammatory tissue, the growth takes place immediately, but it is very slow. No pus is formed from the bacilli of tuberculosis, but the germs grow, and as surely destroy this new inflammatory material as did the germs of suppuration. Then we say that this new inflammatory material has become diseased, that we have a tubercular inoculation or a tubercular focus of disease. So we see there is a vast difference, as I define it, between inflammation and disease, that injury may have been produced by a trauma or embolism; in any case the rapid effort of nature to repair the injury which has been done builds up new inflammatory material, and into this material is inoculated the germs which are floating in the circulation.

Should these germs be pyogenic, then a very rapid osteomyelitis with the formation of abscess is almost sure to take place. If, on the contrary, the germs of tuberculosis are floating in the circulation, and they come in contact with this area of normal inflammation, then so surely will the diseased condition be a tubercular disease of the spine, which begins insidiously, progresses slowly, and produces destruction by ulceration or caries. If absorption has taken place from the Pyerian patches, and inoculation takes place into a focus of new inflammatory material, then we have what is known as the "typhoid spine or hip." The same may be said of actinomycosis. After inoculation has taken place with the germs of tuberculosis, the growth in this new inflammatory material is very slow. After a time, the entire new material is destroyed. Just outside this area of disease a new barrier has been thrown up by nature—a new barrier of inflammatory material. Into this the tubercular germs rapidly grow, and they destroy this new wall of inflammatory material, and so the normal process of repair goes on just a little in advance of the disease.

Tubercular disease never produces an abscess per se. We all frequently find large tubercular cavities which are filled with tubercular materials, but pus is absent. But just as soon as one of these cavities become inoculated with pyogenic germs, then abscesses form immediately; so that it may seem a little heterodox for me to say that abscesses of the spine are seldom prevented by any treatment, after inoculation has taken place, and that when inoculation of the pyogenic germs takes place into an old focus of tubercular disease, that case will rapidly go on to the formation of abscess in spite of anything that can be done, with an occasional exception, and depending upon the physical condition of the patient. By this I do not mean to say that proper treatment is to be discouraged, because the sooner the tubercular focus of disease is done away with, the sooner will the powder magazine be removed from the patient.

We see, then, that these cases of Pott's disease of the spine are always preceded by a lesion in the bony structures, which lesion is either produced by trauma, embolism, or some other pathological cause, producing an area of inflammation into which inoculation takes place from the germs which are floating in the circulation. The condition is purely local, and has nothing whatever to do with a "constitutional taint." The reason why one child is affected with local tuberculosis of the spine, and another is not, is to be found in the condition of the child. That condition is known as struma. Struma is not a disease; it is a condition, and I hope that I never will see again printed the terms "strumous joint" or "strumous spine." Struma probably exists in the protoplasm of the ultimate cell, and measures the resistance of that protoplasm to the attack of germ life. Scrofula we now know to be tuberculosis, so that "scrofulous spine" and "scrofulous joints" have been relegated to the obscurity which they so justly deserve. General tuberculosis takes place as the result of multiple inoculations in different parts of the body from absorption.

The pathology of the affection depends, of course, entirely upon the etiology. In discussing the etiology I have touched somewhat upon the pathology, so that can be passed over briefly. It is needless for me to say that the disease is, as a rule, with an occasional exception, located in the bodies of the vertebræ or between the bodies of the vertebræ in the intervertebral cartilages, but more frequently in the bone itself. As I have already said, I will not enter into a minute pathology of this condition, because space will not allow; instead, I will present some cuts of pathological specimens, which will explain the location of the disease and will represent closely the conditions found in this affection.

TREATMENT WITH A PROPER SPINAL SUPPORT.

Fig. 1 is a cut of a specimen taken from a case of Pott's disease of the spine. It shows how sharpely bent is the spinal canal, yet the cord has not been pressed upon. It also shows the consolidation that has taken place.

Fig. 2 is another photograph of a vertebral column which shows that the body of one of the vertebræ has been destroyed, and the portion of one of the vertebræ has been pushed into the canal against the cord. I show you these cuts from photographs to prove that there is no invariable rule in these cases—that there are many exceptions.

Here is still another cut (Fig. 3), showing the bodies of two vertebræ destroyed. By pressure the canal has been absorbed posteriorly, thus giving plenty of room for the spinal cord. This is a very interesting fact.

In regards to spinal bracing, I find that the aluminum corset is the best support that can be made. (See Fig. 4.)

I offer it not as a substitute for many of the braces and corsets now worn in the acute forms of Pott's disease and lateral curvature. I suggest it rather to take the place of such braces in cases requiring permanent bracing, or in individuals who are desirous of securing a support at any time which combines durability with lightness and comfort. So soon as a case of lateral curvature has been arrested, or the greatest amount of benefit has been derived from treatment, the aluminum corset will then be found a most agreeable permanent support.

Fig. 1.

Fig. 2.

Fig. 3.

Fig. 4.

The aluminum corset has these qualities to recommend it to the patient: (1) lightness; (2) durability; (3) it is thin and does not interfere with the form and clothing; (4) being extensively perforated makes it the coolest and most agreeable of supports; (5) the patient can wear it during bathing.

An ordinary corset weighs from one to two pounds, depending on the size. To prevent cracking and to protect it from perspiration it is covered with a waterproof enamel which is applied by heat.

### II. Hip-Joint Disease.

Before considering the early symptoms of hip-joint disease, I would like to call attention briefly to a few facts which are observed clinically. Joints attacked by inflammation either intra- or extracapsular, have a condition of rigidity or spasm of the muscle about them. This is due to irritation of the terminal nerve plates in the area of disease, transmitted through the reflexes. The muscles operating upon the joint, which are supplied by a nerve given off from a common nerve trunk (one branch distributed to the area of the disease, the other to the muscle) are affected by spasm, while the other muscles may remain quiescent. That muscles affected by spasm will rapidly atrophy is well known. These facts are observed particularly in inflammation of the knee-joint. The knee-joint is supplied posteriorly by branches from the great sciatic nerve. The patella is supplied by nerves given off from the anterior crural. When inflammation attacks the condyles, flexion and rapid atrophy always takes place, but in patella disease, or disease located anteriorly, the limb remains in the straight position, owing to the fact that the reflexes are distributed through the anterior crural and not through the great sciatic. Assuming that these propositions are correct, and clinical observations seem to demonstrate them, we must at once conclude that rigidity of the muscles from spasm, producing a limit of motion, would be the first symptom observed in any joint disease. Limit of motion in any joint produces deformity. We would designate as the second most common early symptom in joint disease-deformity. This limit of motion and deformity produces a limp. So I think we can safely say that limit of motion, deformity, and limp are nearly always, if not always, present in hip-joint disease in the early stages.

There are in general joint diseases eight cardinal symptoms, two or more of which are always present. These cardinal symptoms are pain, heat, swelling, pain on joint pressure, limited motion, spasm of the muscles, atrophy and deformity. Each joint has superadded to these eight cardinal symptoms other special symptoms. These special symptoms are due to the anatomical characteristics of the joint. In hip-joint disease pain is not always a common symptom; rise of temperature, owing to the depth of the joint, is hardly perceptible; swelling is not seen until effusion or dislocation takes place; pain on joint pressure is present only in intracapsular disease, located between or near the articular surfaces. Limited motion, spasm of the muscle, limp and deformity, with apparent lengthening or real shortening are nearly always seen associated together. Atrophy pretty constantly occurs, especially in bone diseases, and it may occur as early as the tenth day. The other symptoms observed in the early stages are night cries, pain in the knee, flattening of the buttock, partial or complete obliteration of the gluteal fold.

When the limb is in a straight position the muscles accurately balance it, but when the limb becomes flexed, the action of these muscles is changed in proportion to the amount of flexion. If these muscles are in a condition of excitability or spasm from reflex irritation, one can easily see how various deformities can take place, depending entirely upon the position of the limb when the muscles act. When this great mass of muscles is affected by spasm, which is always the case in inflammation, one can readily see how limit of motion and deformity, to a greater or less extent, must be the earliest symptoms observed.

Before the last American Orthopedic Association, I presented a model, together with several dissections which I had made of the joints, for the purpose of demonstrating why the limb assumes certain positions, with occasional exceptions, when the joint is inflamed. The capsule of the normal joint is twisted around the head and neck in such manner that when the limb is in the straight position, great tension is exerted upon the joint through the capsule and its other ligaments. Now, when the joint or capsule becomes inflamed, the patient invariably places his limb in a slightly flexed and abducted position to relieve tension, and changes altogether the action of the muscles; they being in a condition of spasm, together with the voluntary act, produce the deformity of the first and second stages of the disease. When flexion takes place just a little farther, the action of the muscles is entirely changed; abductors become inward rotators; outward rotators become, to a certain extent, abductors, and the like. Resistance not being offered to the abductor muscles, the limb by their contraction, passes over to the deformity of the third stage of hip-joint disease, that is, abductive flexion and inward rotation. There are exceptions to these deformities, which I have designated as erratic, but they will not be considered now.

These deformities take place whether disease is intracapsular or extracapsular, whether there is effusion into the joints or not; and let me say here that only a limited number of cases have effusion into the joints in the early stages. To conclude, the importance of symptoms, I believe, speaking generally, occur about in the following order: (1) limit of motion; (2) deformity, with apparent lengthening or real shortening; (3) limps; (4) atrophy—bone disease; (5) pain in the knee—with absence of knee-joint disease; (6) pain on joint pressure; (7) night cries, in absence of other joint disease; (8) flattening of buttock, with change in gluteal fold; (9) heat; (10) swelling.

The order of these symptoms might be transposed a little by some authors, but this order will answer for diagnostic purposes.

### TREATMENT.

The treatment of hip-joint disease is divided into the operative and mechanical. In all cases where abscesses are present they should be immediately evacuated. This enables the surgeon to intelligently explore the diseased joint with his finger, and ascertain to what extent the disease has progressed. If the head of the bone is separated from the neck it should be removed together with the great trochanter, and the neck. The acetabulum, if diseased should be thoroughly curetted, together with any other diseased tissue that may be found in the joint. If only small points of disease are found within the joint, those should be curetted, together with whatever diseased tissue exists within the joint, and the cavity washed out with bichloride solution, 1 to 2000. The joint should now be filled with a solution of iodoform and glycerine, one-half of iodoform to four of hot glycerine.

After this has been done the patient should be put in bed, with extension in the line of deformity and lateral traction above the knee, amounting to about three pounds. Day by day the limb should be lowered, until the deformity is overcome. When the deformity is overcome, the lateral traction fixation splint, which I devised and use in the Post-Graduate and other dispensaries, should be adjusted, and the patient put on crutches with a high shoe on the well leg. Pus and tubercular material destroy living tissues and when joints are allowed to macerate for weeks and months in these materials, which now seems to be the favorite method of many of our orthopedic surgeons, extensive destruction of the bone will almost surely follow. In many cases extensive cutting of muscles, tendons and fascia may be necessary to overcome the deformity. The reader will see, then, that we believe that deformities should be first overcome and all abscesses opened before the mechanical work begins. No case of hip-joint disease need recover with angular deformity and to secure and attain this end, steps should be taken at the commencement of treatment to place the limbs parallel, after which the lateral traction fixation splint, already alluded to, will prevent the patient from becoming again deformed.

### MECHANICAL TREATMENT.

Fig. 1.

For many years the profession has been taught that the long traction splint used by Sayre, Taylor and others, was the proper machine to use. The patient is allowed to walk upon this splint, using it as a perineal crutch. The splint stops at the trochanter and exerts no power over the joint on that account to fix the joint. The patient, stepping upon this splint with the strap around the perineum, causes trauma of the joint while walking, and nearly every splint that I have seen adjusted allows the patient to put his toe upon the ground, which of course drives the head of the bone into the acetabulum each time the patient steps (see Fig. 1.) This pumping of the head of the bone backwards and forwards into the joint at the rate of 2,000 times an hour each day, as the child runs, accounts for the disastrous results which we see published from institutions where this splint is used. Angular deformity, which produces shortening, nearly always results from the use of this splint. The statistics published by Shaffer and Lovett, in the New York Medical Journal, from the 59th Street Orthopedic Dispensary, in 39 cases reported on in a series of many hundreds: ankylosis, 19; slight motion, 6; total, 25. Motion from 10 degrees, 7; motion to right angle, 3; motion free, 3.

The three with free motion were treated during the first stage of the disease; two were under three years old. There were only two cases without shortening. The splint used was the long traction, which I have already described—one which admits of free motion at the hip-joint and the patient is allowed to walk upon it. This splint was devised during a time when it was believed that fixation would produce ankylosis of the joint, and that motion was necessary to keep up the nutrition of the joint. It is needless to say that we have outgrown both of these ideas. The statistics of Chambers Street Hospital of 50 consecutive fractures of the elbow joint show only one case of ankylosis. These patients were fixed in plaster-of-Paris for many weeks, without passive motion. In the Post-Graduate Hospital and University Dispensary we fix our cases of hip-joints from one to five years without motion, with the lateral traction fixation splint, and in our large series of cases not one has resulted in bony ankylosis, excepting cases with great destruction of bone; and where we have had control of the patient even such have recovered, without angular deformity.

Fig. 7.

Fig. 7 shows range of motion in a case fixed sixteen months absolutely. Shortening is seen in this long series of cases only from non-development of the limb, and extensive bone destruction. The accompanying cuts and description will convey a very accurate idea of the splint, which we use in our treatment after the deformity has been overcome in bed. Tissues inflamed or diseased should be put at rest, to allow the normal process of repair to take place without the trauma of motion. This is the law. It is applied in the treatment of the iris, fractures, sprains, and any other tissue that can be immobilised. To carry out the requirements of this law in so far as possible, I was led to devise these splints illustrated in this article. To fix the hip-joint, a splint must extend from the foot to the axilla. (See Fig. 2, 3, 4 and 6.)

Fig. 3 represents the perineal crutch with abduction bar adjustible by means of the key, for the purpose of making lateral

Fig. 2.—The Cheap Dispensary Splint.

Fig. 3.—Inside Bar and Lateral Tractions.

extension. The steel bar is adjusted to the steel ring, which makes a firm crutch, the pressure coming on the tuberosity of the ischium. Adhesive straps, extending too near the body from the ankle furnish means of extension by tightly buckling them to the straps, the ring furnishing counter-extension. The rod ending in the upper ring, prevents flexion and extension of the legs. The splint is intended to prevent every motion at the hip-joint, and at the same time apply extension in a line with the neck of the femur. Fig. 4 shows the crutch and splint adjusted, the patient using crutches, and standing upon a high shoe upon the well leg.

This splint I found a little too expensive for dispensary work. I then constructed the splint (Fig. 2) which simply does away with the extension joint and key. This was also too expensive for dispensary work, but both splints did the work perfectly.

Fig. 4.—The Patent Splint, Adjustive High Shoe and Crutch.

Fig. 6.

After a time, for my poor patients in the hospitals and dispensaries, I succeeded in perfecting a cheap splint, which applies the principle of fixation and traction in the line of the neck.

A glance at the cuts will convey the idea. Figs. 2, 3, 4 and 6 are the single and Fig. 5 the double splint for double hip disease. The splint is a bar of steel, extending from the foot to the axilla, accurately bent to fit the body. A tracing made on paper by laying the child on it will assist in shaping the bar.

A pelvic belt, a thoracic belt, and a steel perineal ring complete the fixation part of the splint. The straps in the foot-piece buckle to adhesive straps attached to the leg, which make longitudinal traction. The strap lashes the leg to the splint, making lateral traction precisely as the abduction bar acts in Fig. 3.

An ordinary blacksmith can construct this splint.

Fig. 5.—Double Dispensary Splint.

Fig. 8.

Figs. 8 and 9, illustrate the anatomy of the muscles surrounding the hip-joint. These muscles, when affected by spasm, act in a line parallel with the axis of the neck of the bone, hence the necessity of lateral traction to overcome intraärticular pressure. Fig. 10 shows how comfortably a patient can sit with the lateral traction fixation splint, fully as comfortably as with a long traction splint.

Before this or any other splint is adjusted, however, the patient should be treated in bed until deformity is overcome and the active stage of the disease somewhat modified.

To conclude, my observations lead me to believe that the most serious element of destruction in hip-joint disease is the trauma and pressure produced by the spasm of the muscle; that fixation

Fig. 9.

of the joint without extension is an impossibility; that the successful treatment of the joint must depend upon its absolute immobilisation, which can only be produced by proper extension and fixation; that the constitutional treatment of hip-joint disease amounts to but little, independent of mechanical treatment; that mechanics is everything; that extension in a line with the axis of the shaft and deformity alone, in hip-joint disease, is entirely wrong; that extension should be made in a line parallel to the axis of the neck—in other words, two lines of extension—otherwise the idea of extension is not perfectly carried out; that ankylosis of the joint is not produced by immobilisation, but by the severity and character of the inflammation; that the long traction hip-splints in general use neither properly extend nor immobilise the joint; that intraärticular pressure results in the destruction of the joint or ankylosis in a large percentage of cases is proved by statistics; that the results in hip-joint disease should be as good as those of knee-joint disease, and will be, provided perfect immobilisation can be carried out; that patients should never be allowed to step upon any portative apparatus; that a high shoe on the well leg and crutches should be insisted upon until the patient is cured; finally, that the angular deformity seen in cured cases should not occur, and such cases are a standing rebuke to the splint and methods employed. In other words, no patient with hip-joint disease need ever recover with angular deformity. In exceptional neglected cases of dislocation a slight amount of deformity had better be left than resort to osteotomy.

Fig. 10.

HOW THE ORTHOPEDIC SURGEON SHOULD TREAT TUBERCULAR AND PURULENT ABSCESSES.

In regard to abscesses, I desire to present to you the latest ideas in regard to the etiology of the abscesses, and how the orthopedic surgeon should treat them.

Abscesses cannot occur excepting they are preceded by inflammatory action; that is, a lesion must occur first, and then the reparative process which we call inflammation—that is, a wandering or proliferation of cells; building up of new inflammatory material constitutes the area of normal inflammation. As I have already stated, this normal inflammation is produced by trauma, by anything which will injure the tissues; anything which will excite the reparative process, the process being inflammation. At this time there may be floating in the circulation the germs of suppuration, the streptococci, etc., or the germs of tuberculosis, which, coming in contact with embryonic germs, cause infection. If the process of repair goes on uninterruptedly tissues are restored, but the very moment infection occurs, a new barrier of inflammatory material is thrown up around it, and this cavity may be filled with tubercular material. Subsequent inoculation may take place by the introduction of pyogenic germs, and then we have a tubercular and purulent process. This material lying in contact with tissues, destroys them, except where the fibrous sac forms, known by the older authors as a pyogenic sac, and when it does, it builds up a barrier around this mass of pus or tubercular material, and the abscess becomes encysted. If burrowing takes place in any direction by the corroding and infecting of tissue, the breaking of abscess into cavities and pus burrowing will be the result. If the absorption takes place, and pus or tubercular material is eliminated through the circulation, there is likely to follow disease of the kidney and liver.

These abscesses are treated in a variety of ways, and I will briefly note each of the methods and the reason why we treat them in different ways. If an abscess occurs in the palm of the hand, which is designated as a surgical abscess, it is likely to follow the sheaths of tendons, and an incision has to be made in order to evacuate the pus. If it occurs in the wrist joint, it may rupture anteriorly and follow the sheaths and tendons. If an abscess burrows and discharges through the capsule anteriorly in hip-joint disease, it ruptures directly into the iliacus internus muscle, finding its way through that muscle into the pelvic cavity and there produces its destructive changes.

*Aspiration.*—The surgeon has virtually discarded the aspirator in the treatment of abscess. He no longer aspirates for empyema, and I can understand perfectly how aspiration of a psoas abscess might be good practice, but, as a rule, is bad. Aspiration might be desirable in small children, but as I have previously said, as a rule, in surgery the aspirator has been discarded in the treatment of abscesses. The Germans have a method of injecting them with a solution of glycerine and iodoform, and by means of this treatment some brilliant results have been reported.

When I look over the literature on the subject, and examine the cases which have been exhibited from time to time in which this treatment has been resorted to, I am not altogether in favor of the injection of abscesses indiscriminately. Upon the whole, I would not think of injecting a joint, if it was extensively diseased, with iodoform and glycerine. I would advise by all means, simple puncturing and intelligent exploration with the finger. If the abscess cannot be curetted and all the diseased tissue removed, it is better not to incise the abscess extensively. It is better to puncture and insert a drainage-tube. I would lay down the rule that abscesses and sinuses should never be curetted where there is dead bone at the bottom of them. In those large abscesses where you cannot remove the disease, do not make a big denuded surface. Puncture, establish drainage and wash out the abscess cavity with a solution of bichloride of mercury 1 to 2,000. In cases where the abscess appears externally, make a free incision, curet the cavity, close it up, and primary union of the external wound will result. It would be bad practice to put a scoop into a psoas abscess, because the diseased bone is not removed; it is beyond where we can reach it as a rule. Wash out the cavity as thoroughly as possible, insert a drainage-tube up to the point of the disease, carry the drainage-tube on the end of a stiff probe up to the diseased vertebræ. Following this line of treatment, I have observed that patients do well. Unless you can remove all of the diseased tissue do not curet. Puncture, wash and drain.

If we find when we cut down upon a joint that it is extensively diseased, excision, of course, may be necessary. In all abscesses of joints, which do not show a tendency to absorb, which seem to be tubercular, or joints in which the capsule is ruptured, or the abscess is burrowing, I have freely incised, and I think all these cases should be immediately operated upon for two reasons. In the first place, a small incision is made to introduce the finger, and after introducing it you can intelligently explore and ascertain the amount of disease or pressure. If you find the disease is extensive, then excision may be necessary or erasion, or whatever procedure we decide upon after we have entered the cavity. I think in cases of all joint abscesses, as a rule, we had better operate, except possibly in those which cause no disturbance, and do not increase in size, and I am now operating on all.

TREATMENT.

After the excision is completed, or the abscess has been thoroughly curetted without an excision, in either case the joint should be thoroughly injected with pure carbolic acid which is allowed to remain in the cavity from one to two minutes, after which the carbolic acid is washed away with pure alcohol, and finally the cavity washed with a 2 per cent. solution carbolic acid. A glass drainage-tube, as large as the wound will take, is inserted down to the bottom of the cavity. The tube should be just long enough to be flush with the skin, and through it the packing is done, instead of packing the wound as in former practice. This large glass tube acts as a speculum through which at subsequent dressings observations can be made of the condition of the wound and deeper structures. It holds the soft part away and gives the bone an opportunity to granulate up until the tube is pushed out, when a smaller and shorter tube can be used. I look upon this use of carbolic acid and the large speculum as one of the most valuable advances we have made in the treatment of abscesses during the century. Abscesses abounding in Scarpa's triangle should never be opened at that point. An incision should be made along the femur, just below the great trochanter, and carried down to the neck of the bone where the abscess can be observed at this point. This gives the surgeon an opportunity to use his finger for exploration, and if excision is necessary, his incision is in the right place. Knowing that a child lies upon its back twelve hours out of the twenty-four, we can readily see that incision in Scarpa's triangle would not admit of drainage, and burrowing of pus always attends the opening of these abscesses at that point.

To get a clear idea of the pathological conditions present, I have prepared several pathological specimens selected from a large number of photographs to illustrate the subject, and showing the results of abscess or of tubercular maceration of bone.

The reasons why we operate are: (1) to save years of treatment and suffering; (2) to intelligently explore; (3) to get rid of foreign material by drainage; (4) to avoid ameloid disease of the liver and kidney by infection; (5) to prevent burrowing into important structures, such as the peritoneum. I have seen a psoas abscess work into the peritoneum, into the bladder, and into the pelvic cavity. I have seen the quadriceps group of muscles destroyed by the burrowing of pus; (6) to prevent destruction of bone by maceration which leads to extensive destruction; (7) to remove sequestra if present; (8) to apply the scientific rules laid down both by the surgeon and the pathologist.

When to operate. When the capsule is ruptured in rapid osteomyelitis with joint destruction. When abscesses appear at points where they must burrow to other tissues and cavities. For instance, an abscess rupturing anteriorly, in hip-joint disease, discharges into the iliacus internus muscle. All tense abscesses should be operated on. In the presence of dead bone we should always operate. We should operate on rapidly increasing abscess and in joints filled with purulent and tubercular material. We should operate in cases of repeated accumulations or multiple abscess. (9) We should operate in feeble children always; (10) in rise of temperature; (11) when in doubt and for diagnostic purposes.

WHEN TO OPERATE.

First—Always when the capsule of the joint is ruptured.

Second—In rapid osteomyelitis, or dual inoculations with joint distension, causing pain.

Third—Abscesses appearing at points where they may burrow to other important structures, and to cavities, *e.g.*, anterior rupture of the hip-joint; the discharge in such cases takes place into the iliacus internus muscle, and at once burrows into the pelvic cavity. Tubercular or purulent disease of the wrist-joints; discharging among the flexor or extensor tendons, destroying, in such cases, if left to nature, the muscles of the forearm and the tendons in the palm of the hand. Large abscesses in Pott's disease of the spine, which are likely to rupture into the peritoneal cavity, the bladder, rectum and pelvis of the kidney. Retropharyngeal abscess in cervical Pott's, which should always be opened externally to prevent infection of the lungs; fluctuating tumors in dorsal Pott's, which are likely to penetrate the pleural cavity or mediastinum; rupture of the capsule of the knee-joint superiorly, the abscess discharging underneath the quadriceps group of muscles; the same is also true when burrowing takes place from any joint. In ankle-joint disease, where the abscess is ruptured posteriorly burrowing upwards behind the tendo-achilles, finding an outlet often three inches above the joint; such joints cannot drain, owing to the location of the sinus.

Fourth—All tense abscesses, because one can never tell in what direction burrowing will take place.

Fifth—Always when carious or necrosed bone is present. Inasmuch as this can never be determined until the abscess is opened and intelligently explored, I believe that nearly all abscesses should be opened and intelligently explored with the finger, to determine this most important complication ; certainly, in all abscesses where the presence of dead bone might be suspected.

Sixth—Rapidly increasing abscess.

Seventh—Joints filled with tubercular or purulent material. Drainage at the lowest point and the washing out of the joint is certainly a rational scientific procedure.

Eighth—Repeated occurrence of abscess and multiple sinuses.

Ninth—Abscesses in feeble children, with rise of temperature.

Tenth—In every abscess with rise of temperature.

Eleventh—When there is doubt as regards diagnosis. It is safer to explore and drain than to leave the condition to nature.

In my opinion, it is the best practice where abscess is present in the joint, to follow up the case with appropriate treatment. When it is impossible to determine the amount of disease that is present, or where the abscess is located, it is better practice to operate on the abscess and intelligently explore it. I have put my finger into a joint and found abscess not only in the joint itself, but external to it. I found the joint diseased and a focus of disease located in the epiphysis.

A case occurred in the Bellevue Hospital, and I secured the specimen from the University. The man had on him the mark of a perineal strap and other evidences that he had worn a hip splint of some description. He lay upon the table with his thigh pepper-boxed with sinuses, and when the joint was finally dissected out, one of the students presented the specimen to me; I made a section of it, and have the photographs of the specimen here (see Figs. 7, 8.) This man had suffered from intracapsular fracture. He was thirty years old. The head of the bone became consolidated in the acetabulum, with the neck of the bone in the new articulation, which had been formed by the great trochanter, lying against the neck of the bone. I found a normal articulation, as you will see, between the neck and great trochanter. I found the head of the bone was consolidated in the acetabulum. Cases of this kind should be operated upon and the finger introduced. With the curet used a few minutes one could cure such a case. The prolongation of treatment would not do any good, and the patient might die of infection, as this one did.

In another case, treated by one of my colleagues for several months, I proceeded at once to follow the sinuses down, and found that the abscess had burrowed along up the radius and ulna, and something like four inches of bone were destroyed. I immediately removed all of the diseased tissue, several inches of bone, shortened some of the tendons, and got a fairly good hand. If this case had been operated on early it would not have been necessary to remove four or five inches of the radius and ulna.

Recent examinations have proven that 90 per cent. of all abscesses which at first might have been tubercular were purulent. In other words, a dual inoculation had taken place. From the fact that 90 per cent. of so-called simple tubercular abscesses sooner or later become purulent they should be drained early.

62 East 34th Street.

### DISCUSSION.

Dr. Roswell Park.—Those of us who know Dr. Phelps will not be surprised at anything in the way of force or incisiveness of treatment displayed by him in the discussion of any subject. I think his paper is exceedingly characteristic and proportionately helpful to all who have heard it. He has alluded to the distinction between tubercular disease and rheumatism, and it is very necessary to insist on this distinction because, even today, I get very few cases of joint tuberculosis in my clinic that have not at some period of their history been considered rheumatism; moreover, one-half the cases are stigmatised as such when they come to me. No distinctively rheumatic lesion ever suppurates. Yet cases come to us riddled with suppuration that are still called rheumatism. In the same way cases of acute osteomyelitis are mistaken for rheumatism. Even when bone destruction and spontaneous opening have occurred, they are still called rheumatism. No purely rheumatic lesion suppurates; it takes a secondary or mixed infection to produce pus formation. As I was noticing the doctor's charts and illustration, I could not help thinking of the length of time it has required to get the pathology of tubercular joint disease into the minds of the American profession. Twenty-five years ago, when I was graduated, we had a teacher who made the positive assertion that tuberculosis never occurred in bone; and I made a notation to that effect in one of my text-books. That was a fair sample of the best understanding of the subject at that time. I shall never forget the profound impression made on my mind about twenty years ago, when the American Medical Association met in this city, I being here as the youngest delegate, by Prof. Henry Smith's paper, in which he called attention to the fact that Nelaton had distinctly described all the lesions which we know today as those of bone tuberculosis. A few years later when I could secure Nelaton's paper I found the illustrations equal to anything in my possession or in Dr. Phelps's today, lesions of which the American profession were decidedly ignorant. It has taken a long time to introduce a knowledge of the pathology of this disease among the American profession and Dr. Phelps has done great service by insisting on clearer notions regarding its pathology. With what he has said I can find no fault, and have no occasion to differ.

With regard to treatment, he has laid down indications when one should operate and when one should not. I have been in the habit of telling my students that I did not wish to be understood as teaching that there was one surgery for the rich and one for the poor. I believe that tuberculosis may be self-limited, and in time a recoverable disease; that we may have recovery without mu h, if any treatment. At the same time, I believe that to cure a bad, nonoperative case you must have an immense amount of patience, time and intelligence, factors which a certain class of hospital cases cannot command owing to want of money. So I have occasionally permitted myself to operate on a poor child and resect a hip-joint early, when I would not think of doing it in a wealthy patient who could afford endless rest in bed, change of climate, and the like, because I considered it the greatest kindness that could be done to the patient. I think in case of the average child, where the mother is unable to give the necessary care and attention, that it is better to operate early and let the child get well, and thoroughly well, even with some shortening of a movable hip than to subject it to almost endless confinement in bed and braces. I think it is a defensible proposition that in these cases there is one kind of surgery for the wealthy and another for the poor; and one must adapt his devices to the needs and social condition of the patient. I think that the greatest good will be done to the greatest number of hospital clinic cases by insisting on early, rather than late operations.

I might add that, with Dr. Phelps, I believe in the local nature of this disease in the majority of cases; that tuberculosis and the diseases especially characterised by infectious, granulomatous formations, such as cancer, have many points in common; that these diseases in nine out of ten cases are local in the beginning. Moreover, in this we are also confronted with the same unpleasant necessity for repeated operations as in cancer.

Dr. Park then presented a number of cases illustrative of his remarks.

Dr. Bernard Bartow insisted on the necessity for early diagnosis and treatment. In most cases the disease is too far advanced and the most favorable period for attacking it is passed before the case comes to the surgeon. The condition of partial disability, permitting more or less movement of the part, is one of the most efficient factors in the extension of the diseased area. It is only among the more intelligent people that the condition is recognised and referred in the incipient stage. It is not uncommon to have the parent make a diagnosis of joint disease, which the physician dismisses too lightly, thus affording opportunity for its further progress. The signs are so distinctive in the early stages that there is no reason for overlooking them by a person of moderate intelligence. Interference with joint function, whether the lesion be in the joint originally or in the bone, manifested by limitation of movement in all directions is the earliest and most characteristic symptom. He considered the bone to be the original seat of infection almost universally, especially in cases of children, and the joint involvement secondary. Physicians should not be so disinclined to believe that moderate impairment of function may be due to tubercular trouble. He should be more on his guard against it than of any other condition that can produce lameness in children. This is particularly striking when we take into consideration the number of tubercular lesions discovered in patients who die of other troubles, tuberculosis not being suspected. He considered hip-joint disease and Pott's disease of the spine as very frequently a secondary infection to a generalised tuberculosis.

Dr. Edward J. Meyer presented cases illustrating conservative treatment, the first being a man who entered the County Hospital two years ago, bed-ridden, with numerous discharging sinuses, some spasm of the psoas, lordosis,—a most pitiable condition generally. He was advised against operation and simply desired to live as long as he could and die in peace. He was placed under the excellent hygienic conditions that obtain at the County Hospital and treated by local scraping, thorough cleaning daily, tonics, and is now practically well with no shortening. He has been advised against anything in an operative way. He was kept in bed with absolute rest and some traction.

Case No. 2 came into the hospital eighteen months ago suffering from tubercular spondylitis in the cervical region. The locality discouraged any attempt at operative interference, so the sinus was curetted without trying to reach the focus. He had been treated with sunlight, hot air and good tonics; was kept absolutely at rest in bed, but no jury-mast. Of late he has been treated by cleanliness and tincture of iodine, and is now in fairly comfortable condition with no active lesion at this time. There is now sufficient sclerosis to support the head without a jury-mast.

In case No. 3, the location of the lesion had admitted surgical interference and the reporter had removed half the body of one vertebra, followed by complete rest in bed and tonic treatment. He had never worn a jacket. He has been in the hospital two years and is making a good recovery.

## Figures and Tables

**Fig. I. f1:**
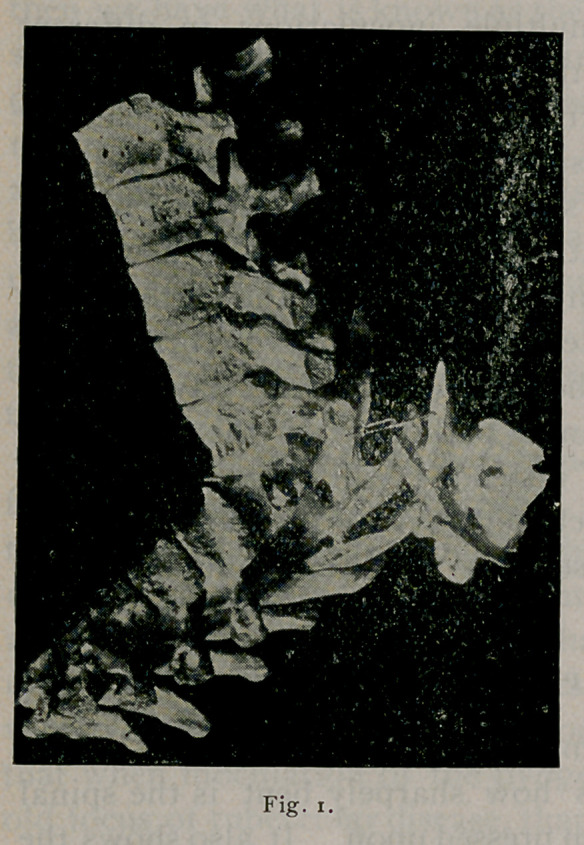


**Fig. 2. f2:**
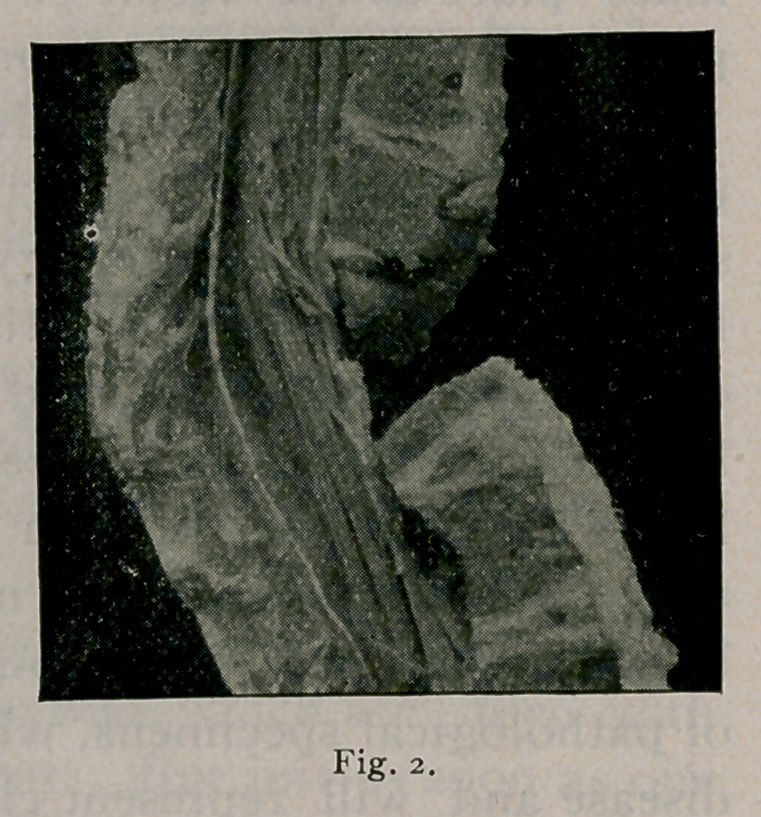


**Fig. 3. f3:**
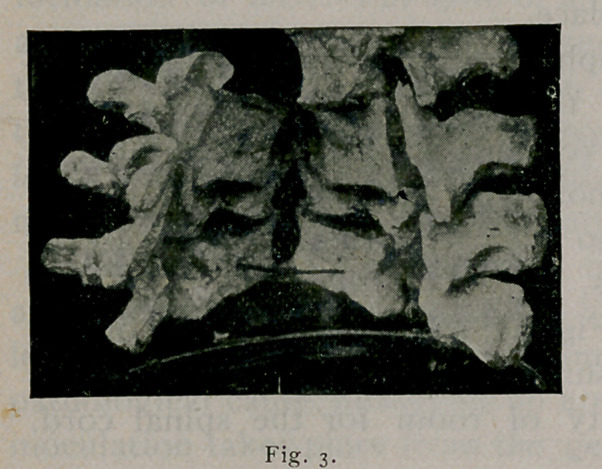


**Fig. 4. f4:**
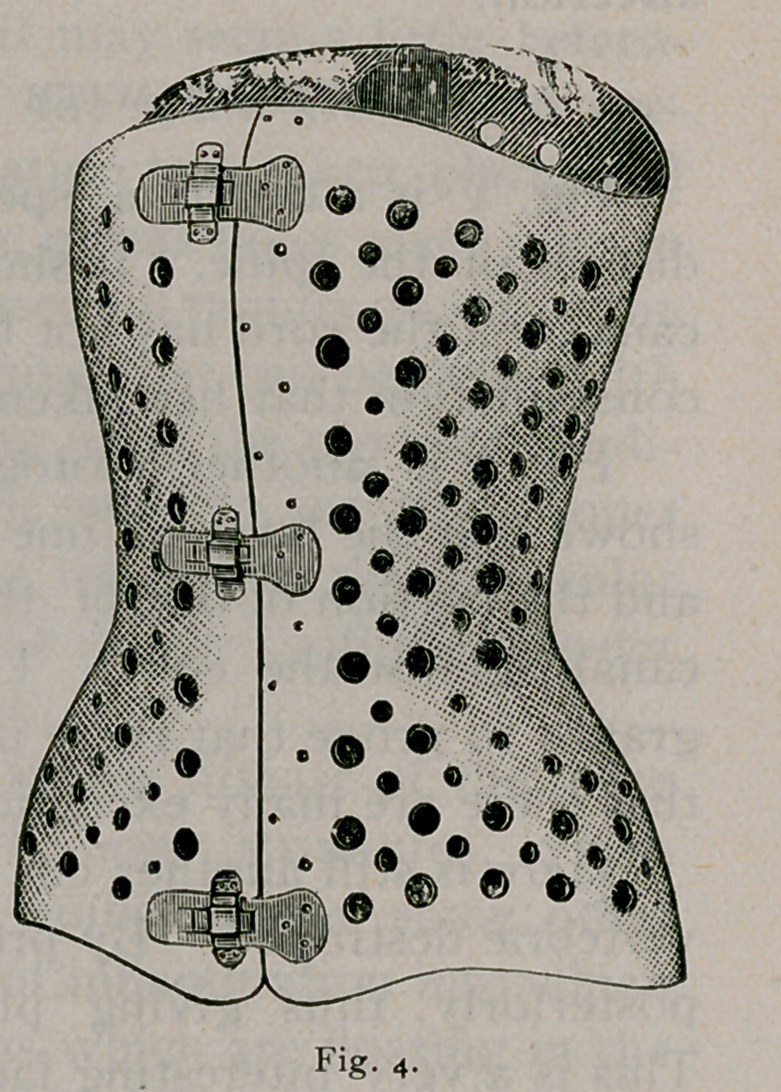


**Fig. I. f5:**
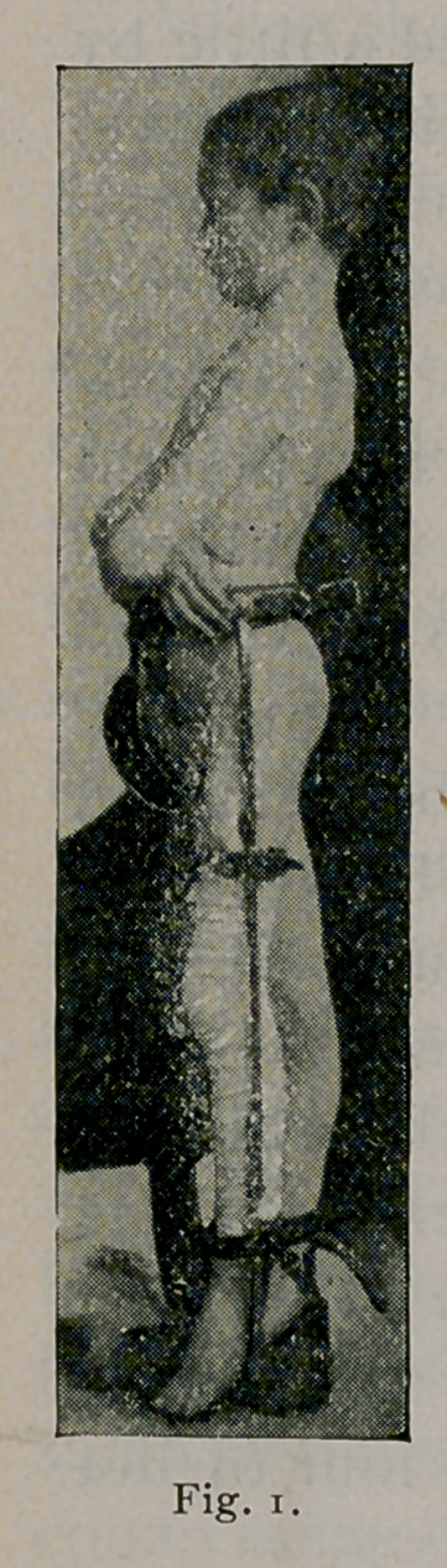


**Fig. 7. f6:**
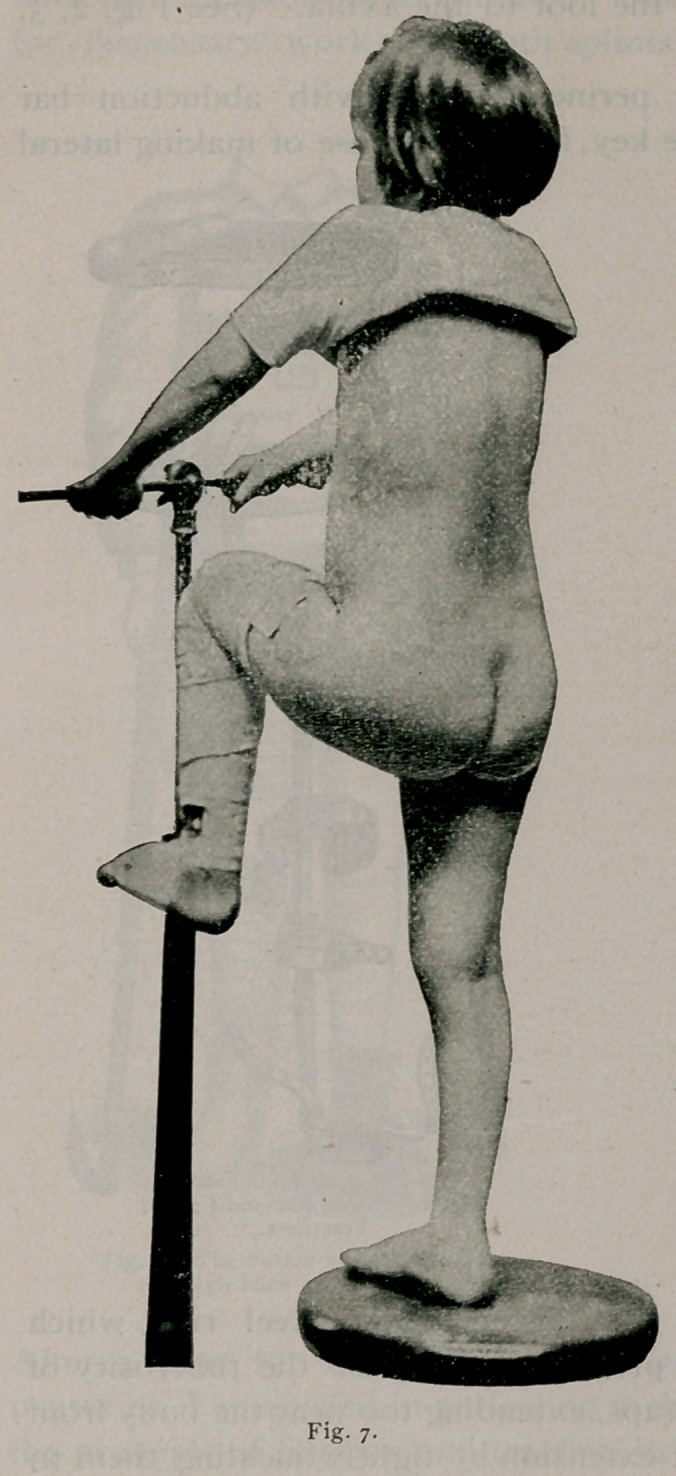


**Fig. 2. f7:**
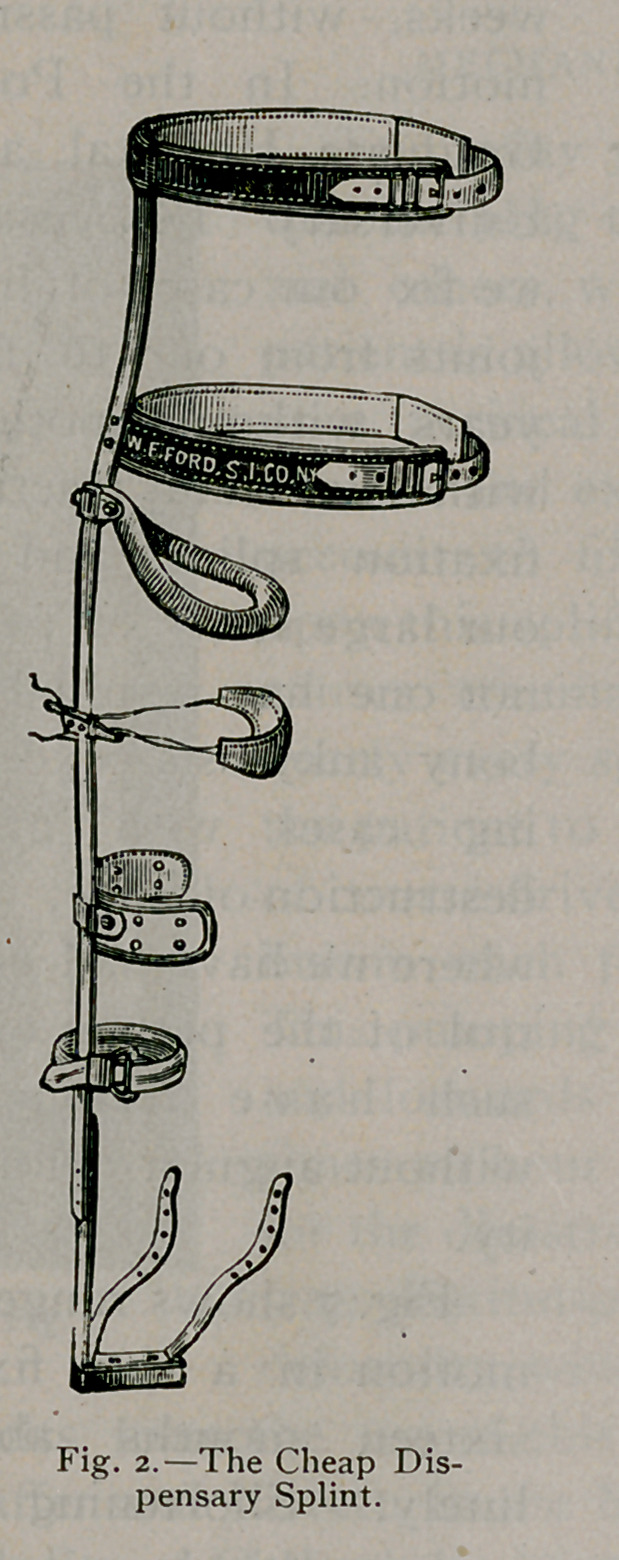


**Fig. 3. f8:**
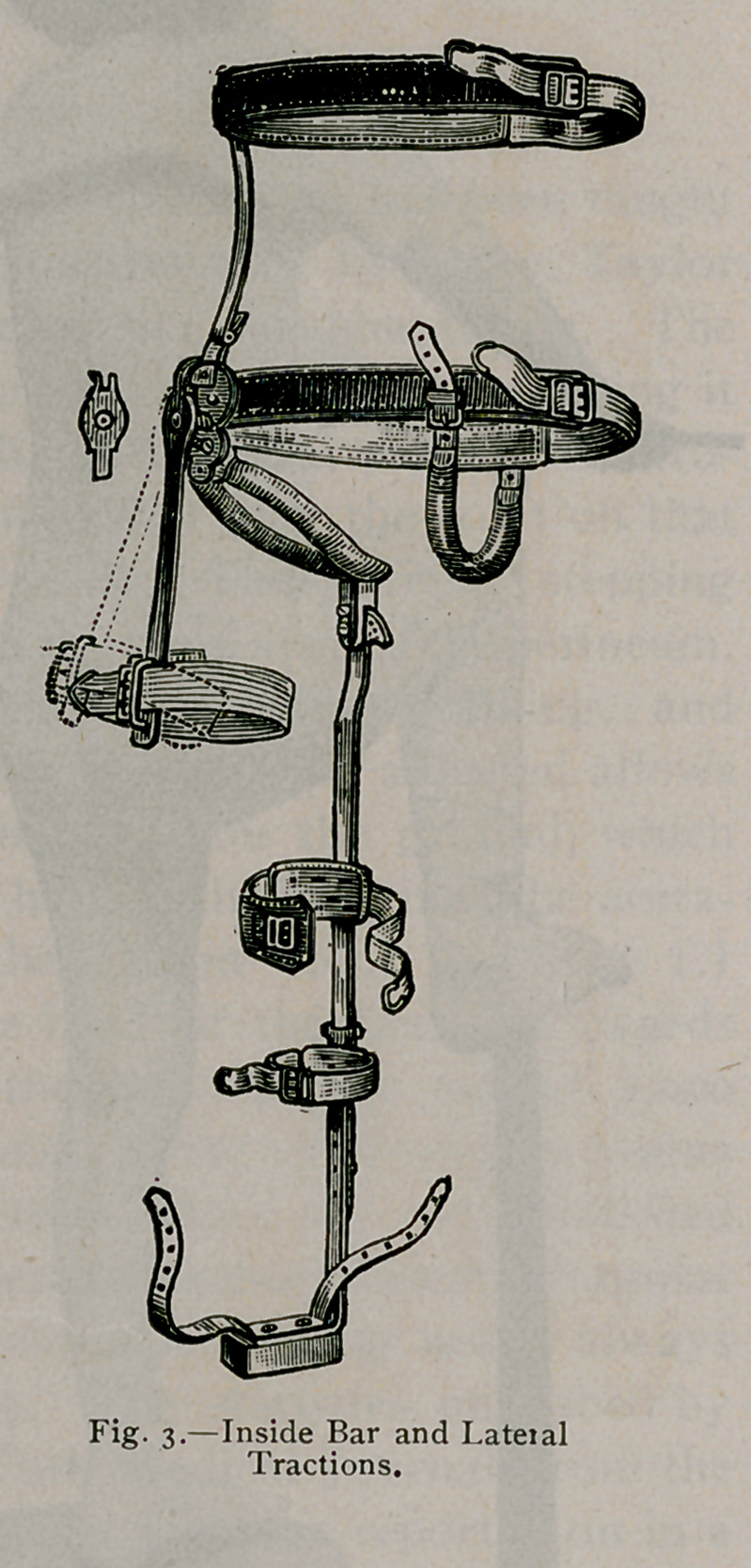


**Fig. 4. f9:**
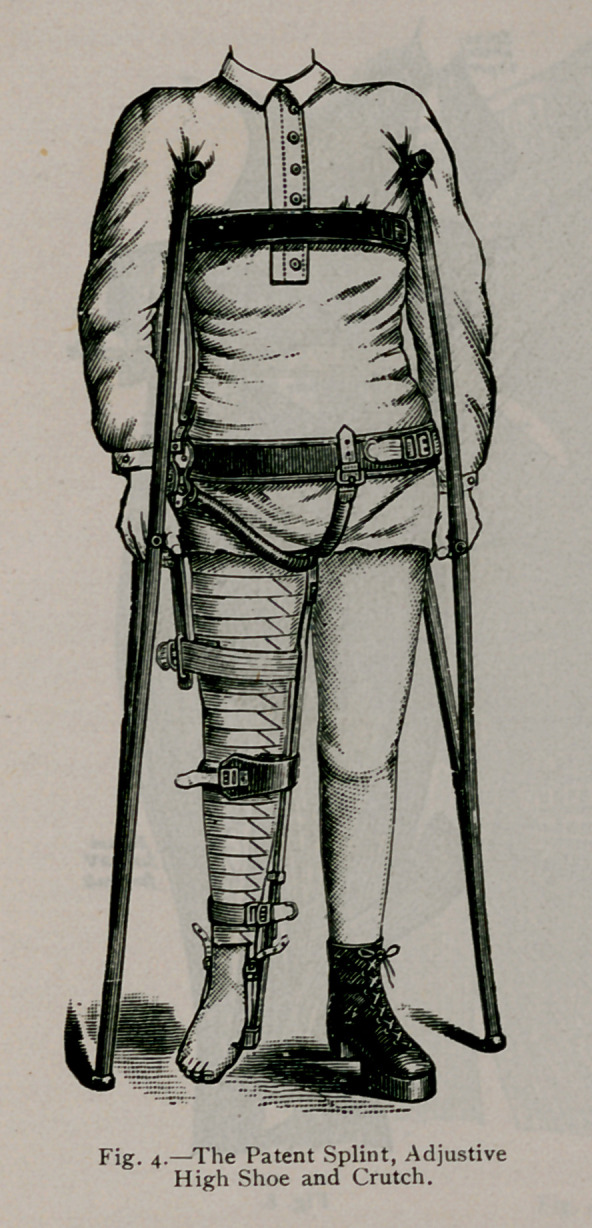


**Fig. 6. f10:**
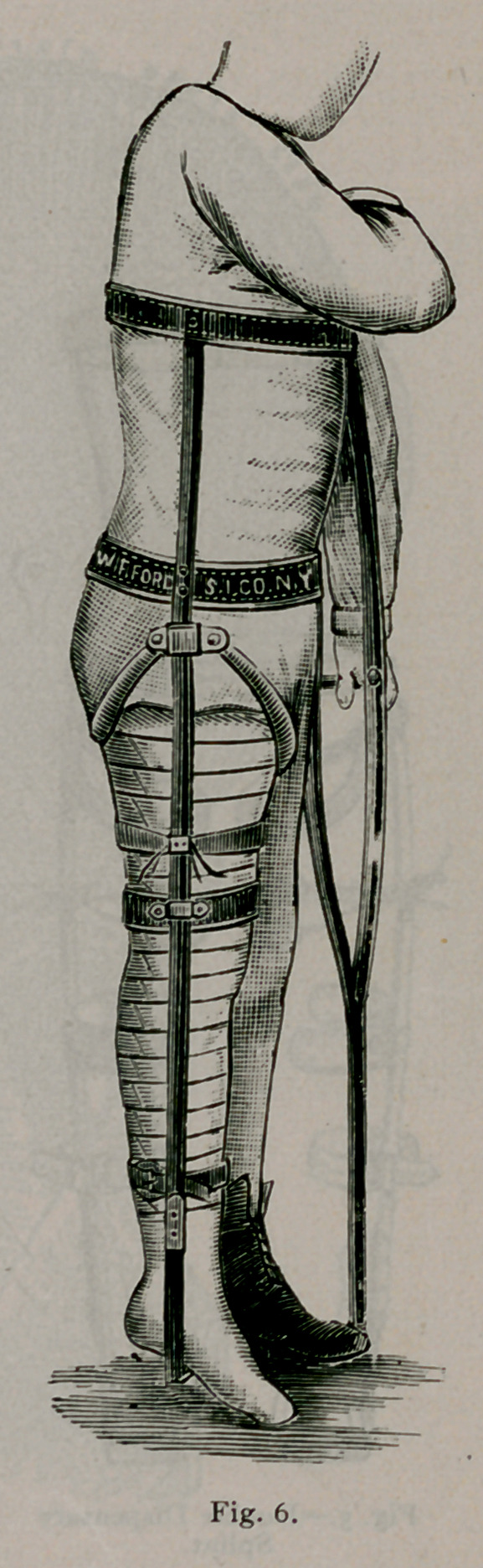


**Fig. 5. f11:**
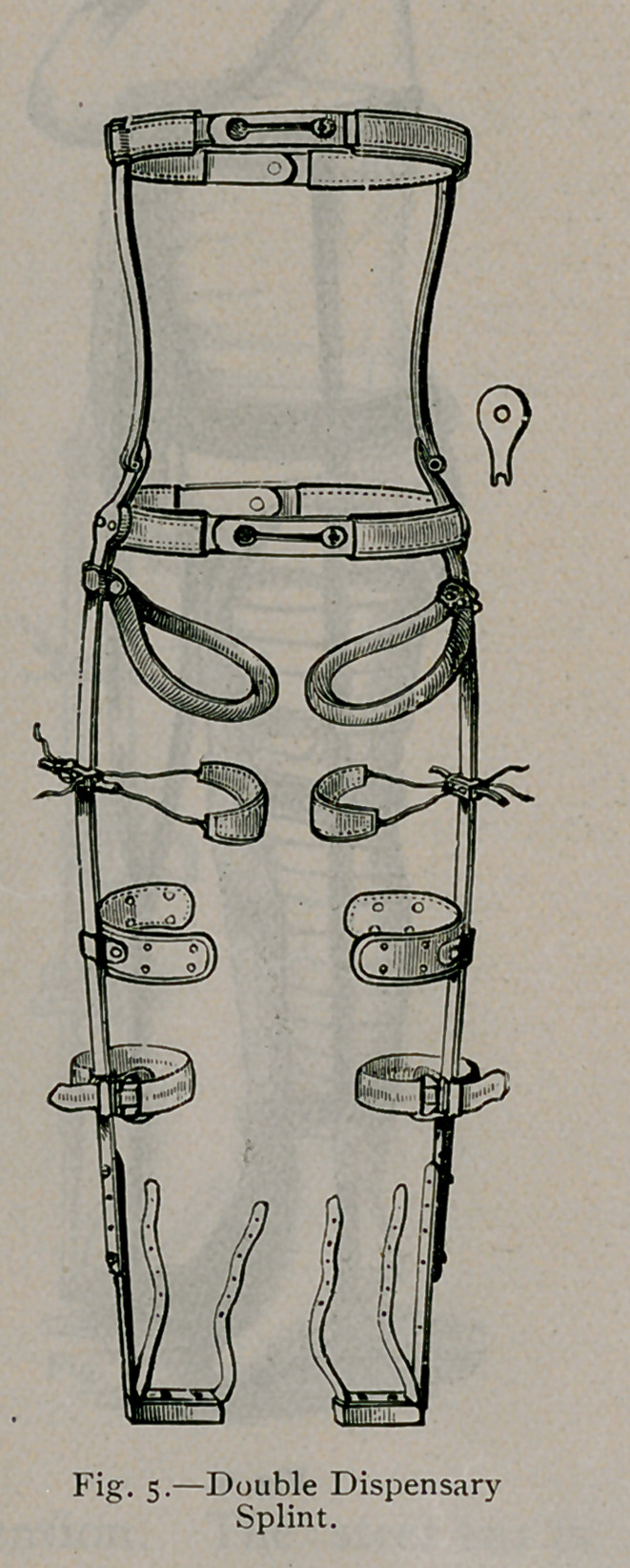


**Fig. 8. f12:**
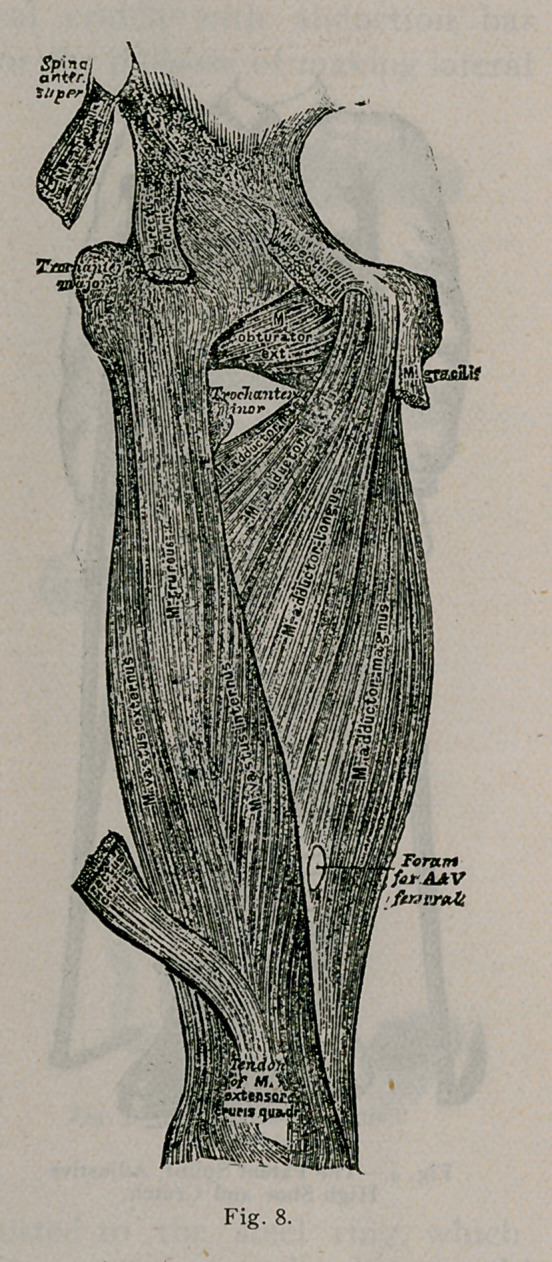


**Fig. 9. f13:**
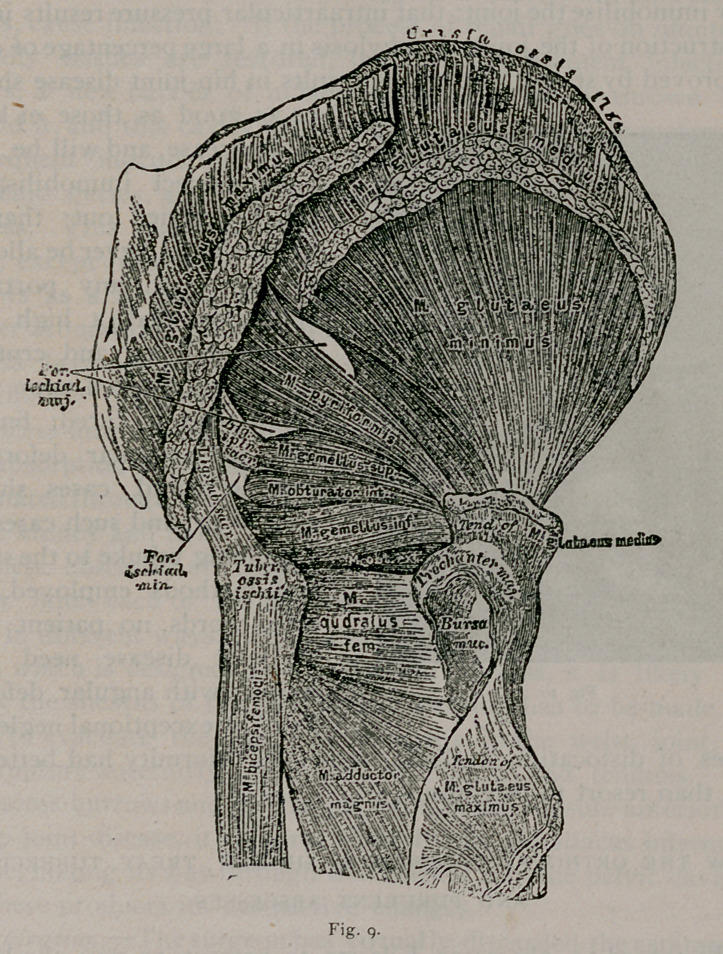


**Fig. 10. f14:**